# Binocular imbalance in patients after implantable collamer lens V4c implantation or femtosecond laser-assisted *in situ* keratomileusis for myopia with presbyopia

**DOI:** 10.3389/fnins.2023.1204792

**Published:** 2023-06-01

**Authors:** Yuhao Ye, Zhe Zhang, Lingling Niu, Wanru Shi, Xiaoying Wang, Li Yan, Xingtao Zhou, Jing Zhao

**Affiliations:** ^1^Department of Ophthalmology and Optometry, Eye and ENT Hospital, Fudan University, Shanghai, China; ^2^NHC Key Laboratory of Myopia (Fudan University), Key Laboratory of Myopia, Chinese Academy of Medical Sciences, Shanghai, China; ^3^Shanghai Research Center of Ophthalmology and Optometry, Shanghai, China; ^4^Shanghai Engineering Research Center of Laser and Autostereoscopic 3D for Vision Care (20DZ2255000), Shanghai, China; ^5^National Engineering Research Center for Healthcare Devices, Guangzhou, China

**Keywords:** myopia, presbyopia, monovision, implantable collamer lens V4c, femtosecond laser-assisted *in situ* keratomileusis, binocular balance, visual function

## Abstract

**Aim:**

To investigate the long-term safety, efficacy, and binocular balance of monovision surgery using Implantable Collamer Lens (ICL) V4c implantation and Femtosecond Laser-Assisted *in situ* Keratomileusis (FS-LASIK) for the treatment of myopic patients with presbyopia.

**Methods:**

This case series study involved 90 eyes of 45 patients (male/female = 19/26; average age:46.27 ± 5.54 years; average follow-up time:48.73 ± 14.65 months) who underwent the aforementioned surgery to treat myopic presbyopes. Data on manifest refraction, corrected distance visual acuity, dominant eye, presbyopic addition, intraocular pressure, and anterior segment biometric parameters were collected. The visual outcomes and binocular balance at 0.4 m, 0.8 m, and 5 m were documented.

**Results:**

The safety index for the ICL V4c and FS-LASIK groups were 1.24 ± 0.27 and 1.04 ± 0.20 (*p* = 0.125), respectively. Binocular visual acuity (logmar) for 0.4 m, 0.8 m, and 5 m were −0.03 ± 0.05, −0.03 ± 0.02, and 0.10 ± 0.03 for the ICL V4c group, and −0.02 ± 0.09, −0.01 ± 0.02, and 0.06 ± 0.04 for the FS-LASIK group, respectively. The proportions of all patients with imbalanced vision at 0.4 m, 0.8 m, and 5 m distances were 68.89, 71.11, and 82.22%, respectively (all *p* > 0.05 between the two groups). There were significant differences in refraction between the balanced and imbalanced vision for patients at 0.4 m distance (for non-dominant eye spherical equivalent [SE]: −1.14 ± 0.17D and −1.47 ± 0.13D, *p* < 0.001), 0.8 m distance (for preoperative ADD:0.90 ± 0.17D and 1.05 ± 0.11D, *p* = 0.041), and 5 m distance (for non-dominant SE: −1.13 ± 0.33D and −1.42 ± 0.11D, *p* < 0.001).

**Conclusion:**

ICL V4c implantation and FS-LASIK monovision treatment demonstrated good long-term safety and binocular visual acuity at various distances. After the procedure, the imbalanced patients’ vision is primarily related to the age-related presbyopia and anisometropia progression caused by the monovision design.

## 1. Introduction

Binocular vision integrates two slightly different images transmitted from each eye to the visual cortex. Effective cooperation between the eyes is crucial for obtaining high-quality images. A binocular imbalance may represent inhibitory binocular interactions by determining the signal strength inequality between the eyes (Kwon et al., 2014). It refers to intermittent partial suppression of monocular vision during binocular fusion (Tao et al., 2022). It shows processing defects in conditions such as autism spectrum disorder (Dunn and Jones, 2020), amblyopia (Mao et al., 2020; Webber et al., 2020), keratoconus (Marella et al., 2021), and glaucoma (Joao et al., 2021), as well as in normal individuals (Xu et al., 2019), which may result from periodic changes in visual cognition caused by neural oscillations in brain activity (Cha and Blake, 2019). Age is a major factor affecting binocular contrast sensitivity, indicating neural processes in binocular interactions (Ye et al., 2023). Further research is needed on the binocular interaction in aged patients, especially those with both myopia and presbyopia. Myopia is one of the most widespread visual impairments worldwide and causes significant disabilities (Wu et al., 2016; Modjtahedi et al., 2021). About one-third of the world’s population currently suffers from myopia, with increasing numbers as the population ages (Holden et al., 2016). The projected increase in the number of older people affected by both myopia and presbyopia implies that society will bear substantial costs (Naidoo et al., 2019).

Monovision is commonly used to treat presbyopia because it is effective and relatively easy to apply in medical practice. It corrects far-sightedness in the dominant eye and near-sightedness in the non-dominant eye (Jain et al., 1996). Monovision refractive surgery is a good alternative for patients with myopia and presbyopia because it improves visual acuity at variable distances (Fu et al., 2018; Luft et al., 2018; Takahashi et al., 2018). However, few studies have assessed the long-term effects of a monovision design, particularly for Implantable Collamer Lens (ICL) V4c implantation and Femtosecond Laser-Assisted *in situ* Keratomileusis (FS-LASIK). Presbyopia progression may continue postoperatively. Furthermore, uneven refractive errors caused by monovision and age-related neural processing deficits may affect binocular balance. This could compromise patients’ visual function and experience, highlighting the need for careful consideration of the potential benefits and drawbacks of this intervention (Yang et al., 2017).

Currently, few studies have evaluated the long-term inter-eye interactions following monovision refractive surgery utilizing ICL V4c implantation and FS-LASIK. Binocular balance and changes in the dominant and non-dominant eyes at different contrast sensitivities remain unclear. Age and anisometropia caused by monovision may be the significant contributing factors (Zheleznyak et al., 2015; Li et al., 2016). Therefore, the binocular balance may be related to presbyopic add and its changes or the intentional target residual myopia of the non-dominant eyes. In addition, it may also be affected by long-term refractive changes after surgery and the visual acuity of the dominant and non-dominant eyes at variable distances.

Thus, this study aimed to evaluate the long-term safety and effectiveness of a monovision design with ICL V4c implantation and FS-LASIK surgery and to assess binocular balance promptly and reliably. It will provide a reference for monovision refractive surgery from an inter-eye interaction perspective. This will help identify and monitor possible defects in inter-eye interactions, enabling clinically accurate evaluation of patient outcomes and prognosis beyond standard visual acuity.

## 2. Materials and methods

### 2.1. Subjects

**Figure 1:**
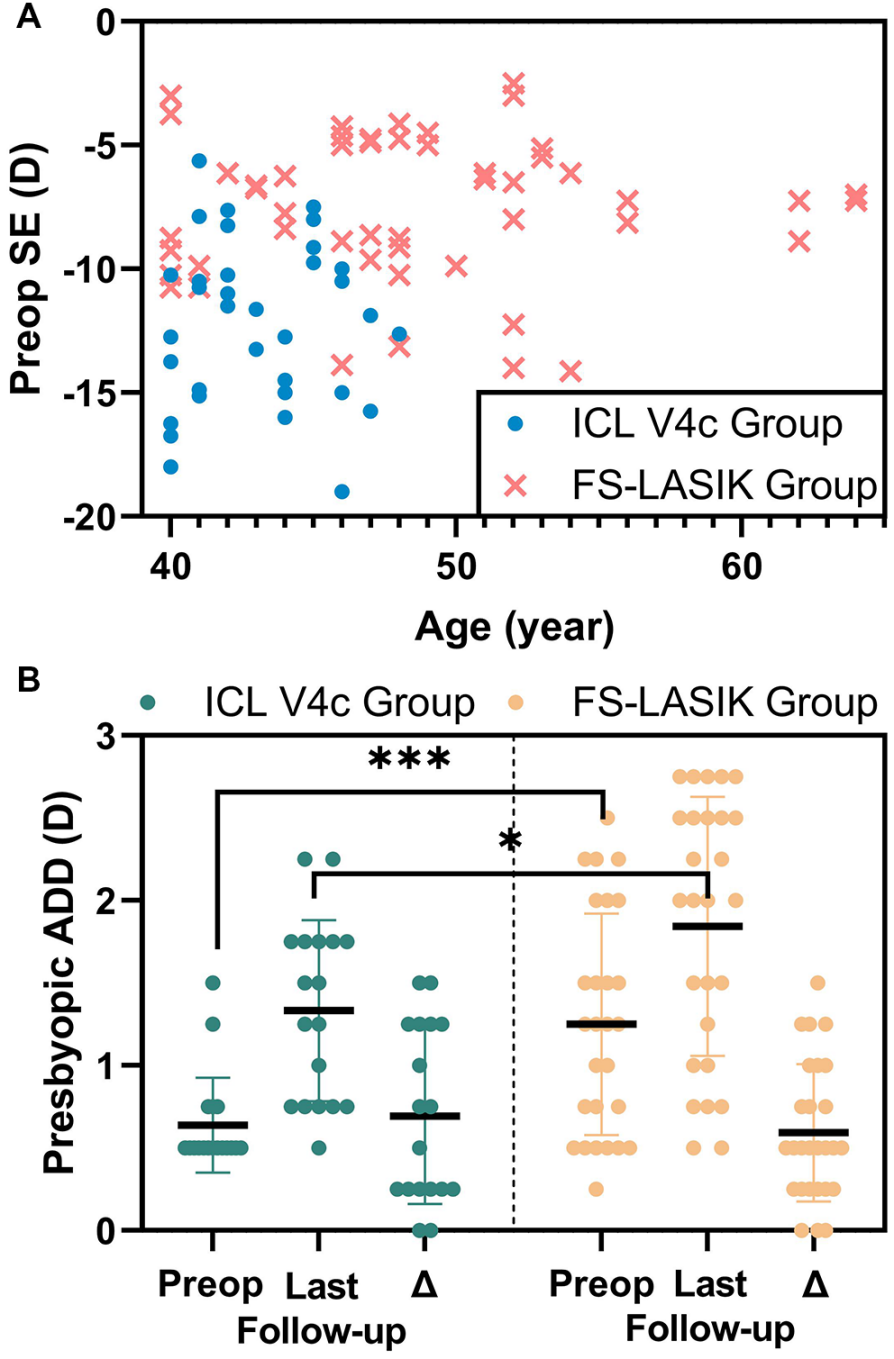
The preop SE and age distributions of the patients **(A)** and the change of presbyopic ADD **(B)** in implantable collamer lens V4c group and femtosecond laser-assisted laser *in situ* keratomileusis group.

This study enrolled 90 eyes in 45 patients (preoperative baseline data for all patients are shown in Table 1). The patient underwent FS-LASIK or ICL V4c surgery for myopia correction and presbyopia at the Fudan University Eye and ENT Hospital between June 2015 and August 2019. Age distribution and manifest refraction are shown in Figure 1A. Preoperative presbyopic ADD and at the last follow-up, and their differences are shown in Figure 1B. This case series study followed the regulations of the Ethics Committee of Fudan University Eye and ENT Hospital (Shanghai, China). It was conducted in accordance with the Helsinki Declaration. All procedures were performed with written informed consent from the patients. The studies inclusion criteria are (1) Age ≥ 40 years or above; (2) Stable spherical equivalent (SE) for the last 2 years (≤0.5D/y); (3) Soft contact lenses should be discontinued for at least 2 weeks and rigid gas permeable lenses for at least 4 weeks before the examination. Exclusion criteria were (1) history of corneal diseases, cataracts, glaucoma, retinal detachment, neurological ophthalmic disease, or other ophthalmic diseases; (2) history of systemic diseases or severe psychological or psychiatric illness; and (3) unsuitable patients for ICL V4c implantation due to endothelial cell density (ECD) <2,000 cells/mm^2^ or anterior chamber depth (ACD) <2.8 mm.

**Table 1:** Preoperative patient demographics.

Characteristic	Mean ± SD	Range	ICL V4c Group	FS-LASIK Group	*p*
Age (years)	46.27 ± 5.54	(40, 64)	43.17 ± 2.48	58.50 ± 7.73	**<0.001**
Gender (male/female)	19/26		6/12	13/14	**-**
Axial length (mm)	27.37 ± 1.96	(23.98, 32.11)	28.69 ± 2.09	26.54 ± 1.33	**<0.001**
Refraction sphere (D)	−8.90 ± 3.69	(−19.00, −2.25)	−12.12 ± 3.23	−7.14 ± 2.80	**<0.001**
Refraction cylinder (D)	−0.88 ± 0.80	(−3.25, 0)	−1.15 ± 1.05	−0.69 ± 0.52	0.161
SE (D)	−9.34 ± 3.78	(−19.00, −2.50)	−12.12 ± 3.23	−7.48 ± 2.80	**<0.001**
CDVA (LogMAR)	0.01 ± 0.08	(−0.18, 0.30)	0.05 ± 0.09	−0.01 ± 0.06	0.002
Dominant eye (OD/OS)	24/21		9/9	15/12	**-**
ADD (D)	1.00 ± 0.62	(0.25, 2.50)	0.64 ± 0.29	1.25 ± 0.67	**<0.001**
K-flat (D)	43.27 ± 1.38	(40.7, 46.60)	43.01 ± 1.59	43.44 ± 1.20	0.122
K-steep (D)	44.33 ± 1.52	(41.10, 47.20)	44.59 ± 1.93	44.16 ± 1.17	0.218

ICL, implantable collamer lens; FS-LASIK, femtosecond laser-assisted laser in situ keratomileusis; SE, spherical equivalent; CDVA, corrected-distant visual acuity; WTW, white to white. Values with statistical significance between ICL V4c group and FS-LASIK group are shown in bold.

### 2.2. Examinations

The equipment used and measured parameters were as follows: (1) An RT-5100 phoropter (Nidek Technologies, Japan) was used to measure spherical equivalent (SE), corrected distance visual acuity (CDVA), uncorrected-distance visual acuity (UDVA), and presbyopic add power (ADD). The safety index (SI) was defined as the postoperative CDVA over the preoperative CDVA, and the efficacy index (EI) was defined as the postoperative UDVA over the preoperative CDVA. D-eyes and nD-eyes were determined using the card-hole method. ADD was measured using the Fusion Cross-Cylinder (FCC) method at a 33 cm distance with optimal distance visual acuity correction. (2) Intraocular pressure (IOP) was measured using a Canon Full Auto Tonometer TX-F (Canon, Inc., Tokyo, Japan). (3) Corneal thickness (CT), anterior chamber volume (ACV), ACD, anterior chamber angle (ACA), and white-to-white ratio (WTW) were measured using Pentacam HR (Oculus Optikgerate Wetzlar, Wetzlar, Germany). (4) Endothelial cell density (ECD) was measured using SP-2000P (Topcon Corporation, Kyoto, Japan). (5) White-to-white (WTW) measurements were performed using IOL Master 700 (Carl Zeiss AG, Germany). (6) Axial length (AL) was measured using IOL Master 500 (Carl Zeiss AG, Germany). Slit lamp and fundus examinations were conducted following pupillary dilation to evaluate lens transparency and exclude fundus lesions.

### 2.3. Monovision design

The D-eyes were targeted for −0.34 to 0.02 D and −1.25 to 0.25 D in the ICL V4c and FS-LASIK groups, respectively. The targeted nD-eyes were around −2.435 to −0.27 D and −2.75 to −0.25 D in the two groups, respectively, according to each patient’s presbyopic add power. In those with planned residual myopic diopters, the target refraction was on trial in-frame glasses preoperatively and was accepted by the patients.

### 2.4. Surgery

The choice of operation was confirmed by surgical indications after adequate communication with patients. For patients who met the two surgical indications at the same time, they could choose by themselves with the knowledge of the two surgeries. The same surgeon (XZ) performed all surgical procedures. The patients were administered antibiotic eye drops four times daily for 3 days before surgery. In FS-LASIK, a 500 kHz VisuMax femtosecond laser system (Carl Zeiss Meditec, Jena, Germany) with a pulse energy of 130 nJ was used for flap creation, followed by a MEL 90 excimer laser (Carl Zeiss Meditec) for stromal ablation, with a pulse energy of 185 nJ. The flap diameter and thickness were 7.5 mm and 100 μm, respectively, with standard 90° hinges and 90° side cut angles. The planned optic zone of 6.45 ± 0.20 mm (ranging from 6.00 to 6.80). After FS-LASIK, a soft contact lens was worn and removed on 1 day postoperatively. Topical levofloxacin, 0.1% fluorometholone solution, and non-preserved artificial tears were administered after FS-LASIK. The detailed steps have been previously described (Han et al., 2019).

The STAAR Surgical online calculator (Version 3.0)^1^ was used to determine ICL power. ICL size calculation was based on the horizontal WTW, ACD, and ATA distances. For ICL sizing, we adjusted the WTW value obtained from the Pentacam and referred to the value obtained from the IOLmaster measurements. In the implantation surgeries, ICL V4c was implanted into the anterior chamber and pre-injected with a viscoelastic agent through the lateral corneal incision under topical anesthesia. After that, the ICL was adjusted using a manipulator, and the viscoelastic agent was replaced with a balanced salt solution. Postoperative antibiotics and steroid eye drops were administered 4 times daily for 2 weeks and tapered gradually. The detailed steps have been previously described (Chen et al., 2016).

### 2.5. Follow-up

The patients were followed up for 4 years, with an average of 48.73 ± 14.65 months. At the last follow-up, the CDVA and UDVA (logMAR) of the D eyes, nD eyes, and both eyes at 0.4 m, 0.8 m, and 5 m were recorded. Measurements were performed using two trial frames and two tumbling E charts (VSK-VC-J 0.4 m/0.8 m, Wehen Vision, China) for the monocular and binocular VA at the distance of 0.4 m and 0.8 m, respectively. A phoropter was used for monocular and binocular distant VA at 5 m.

### 2.6. Binocular balance assessment

Binocular balance was assessed using a modified version of the dichoptic procedure proposed by Tao et al. (2022). The binocular contrast balance task consisted of the use of a sine bar. The images observed by the left and right eyes were divided into three-quarters of a sine function period at a glance y = sin (x)(x = [0, 3*pi/2]) grayscale image, and at a glance y = sin (x) (x = [pi/2, 2*pi]) grayscale image.

The stimulus image was presented on a three dimension (3D) gamma-corrected monitor (LGD2343P, with a resolution of 1920 × 1,080 pixels, the max luminance of 250 cd/m^2^ and a refresh rate of 120HZ). All patients wore 3D polarized glasses to perform the BI tests at a distance of 0.4 m, 0.8 m, and 5 m; each eye was presented with either horizontal or vertical stripes individually, with 100% contrast (Supplementary Figure S1). The same size of stimuli were chosen to control the variables in different distances, because varies pixel size with distances decreases the effect of monovision design. The participants were asked to report whether they saw horizontal stripes, vertical stripes, or a grid. Binocular imbalance, they could not see the black-and-white cross grid. For participants with a binocular imbalance, the level of balance was recorded after reducing the contrast of the image of the dominant eye until the participants could see the grid. Next, the dominant and nondominant eyes of the patient presented with horizontal or vertical stripes at contrast level 1 (100% contrast), whereas the contrast of the opposite eye gradually decreased by 5% each time. The balance threshold range was observed and recorded.

### 2.7. Statistical analysis

All statistical analyses were performed using the Statistical Package for the Social Sciences version 25.0. (SPSS, Inc., Chicago, IL, USA). Results are expressed as mean ± standard deviation. The normality of the data was checked using the Kolmogorov–Smirnov test. Repeated ANOVA was used to compare the pre-and post-treatment and D- and nD eyes normally distributed data, while the paired t-test was used to compare the normally distributed data of the D and nD eyes at the last follow-up, and the Wilcoxon signed-rank test was used for non-normally distributed data. A generalized estimating equation was used to determine the correlation between binocular balance parameters (Balanced/Imbalanced, Level of balance, and range of balance [D-eye and nD-eye]) and refraction parameters (Visual acuity at 0.4-, 0.8, and 5.0 m distances, spherical equivalent, and presbyopic ADD). Differences were considered statistically significant at *p* < 0.05.

## 3. Results

All surgeries and examinations were performed, and no complications, such as cataracts or high intraocular pressure, occurred in any eye throughout the follow-up period. The data loss rate for all the types was <5%.

### 3.1. Safety and efficacy

**Figure 2:**
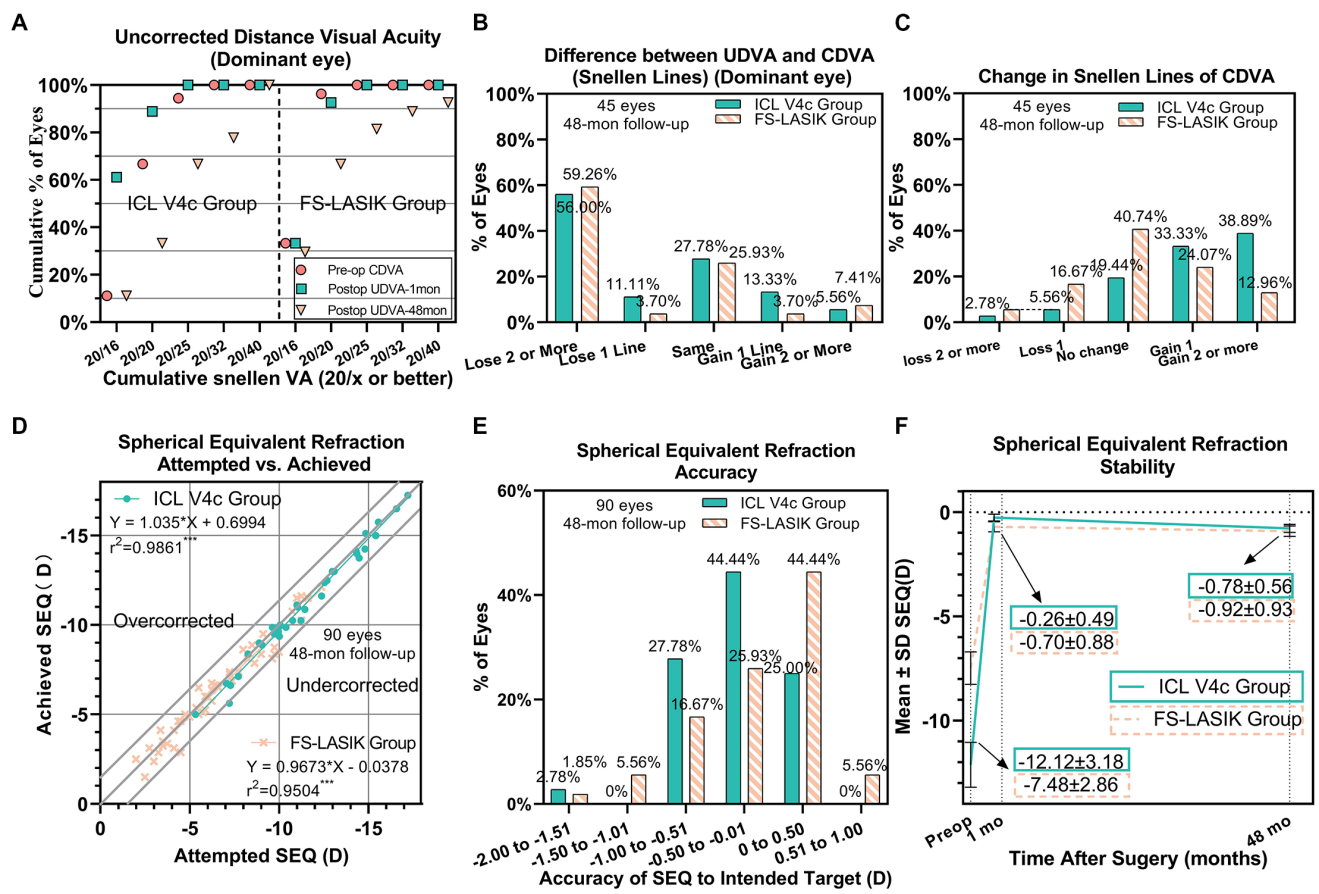
Clinical outcomes of 90 eyes with myopia and presbyopia at the last follow-up after Implantable collamer lens V4c implantation or femtosecond laser-assisted laser *in situ* keratomileusis. **(A)** Dominant eye: Postoperative uncorrected distance visual acuity (UDVA) vs. preoperative corrected distance visual acuity (CDVA); **(B)** Dominant eye: Difference between postoperative UDVA and preoperative CDVA; **(C)** Change in CDVA; **(D)** Attempted spherical equivalent refraction change versus the achieved spherical equivalent refraction change; **(E)** Distribution of postoperative spherical equivalent refraction accuracy; **(F)** Stability of spherical equivalent refraction up to 48 months. D = diopters; Postop = postoperative; Preop = preoperative; mo = month(s).

At the last follow-up, the safety index of the ICL V4c and FS-LASIK groups were 1.24 ± 0.27 and 1.04 ± 0.20 (*p* = 0.125), respectively. Their efficacy indices were 0.77 ± 0.29 and 0.66 ± 0.34 (*p* < 0.001), respectively (Figure 2). The refractive parameter changes in the dominant and non-dominant eyes are presented in Table 2, and changes in the biological parameters are summarized in Table 3. The endothelial cell density (ECD) in the ICL V4c group decreased by an average of 549.42 ± 704.29/mm^2^ (17.14 ± 14.88% and 3.59 ± 3.11% per year). The vault of all eyes after ICL V4c implantation was within the 150 ~ 850 μm range.

**Table 2:** The clinical parameters of the dominant eyes or non-dominant eyes before and after the implantable collamer lens V4c implantation or femtosecond laser-assisted laser *in situ* keratomileusis.

Characteristic	D-or nD-eye	Preoperative			1-mon follow-up			48-mon follow-up		
		ICL V4c Group	FS-LASIK Group	*p*	ICL V4c Group	FS-LASIK Group	*p*	ICL V4c Group	FS-LASIK Group	*P*
UDVA (Logmar)	D-eye	NA	NA	NA	−0.04 ± 0.07	−0.02 ± 0.05*	0.506	0.12 ± 0.12▴	0.07 ± 0.19*	0.501
	nD-eye				**0.03 ± 0.10**	0.22 ± 0.18*	**<0.001**	**0.25 ± 0.17**▴	0.44 ± 0.40*▴	**0.018**
Refraction sphere (D)	D-eye	**−11.11 ± 3.07**	**−6.76 ± 2.36**	**<0.001**	**0.18 ± 0.34**▵	0.03 ± 0.22*▵	0.345	−0.35 ± 0.39*▵▴	−0.09 ± 0.70*▵	0.194
	nD-eye	**−11.97 ± 3.53**	**−7.52 ± 3.19**	**<0.001**	**−0.14 ± 0.41**▵	−1.23 ± 0.83*▵	**<0.001**	−0.90 ± 0.59*▵▴	−1.38 ± 0.73*▵	**0.016**
Refraction cylinder (D)	D-eye	−1.28 ± 1.04	−0.71 ± 0.53	**0.019**	**−0.44 ± 0.33**▵	**−0.17 ± 0.31**▵	**0.027**	**−0.39 ± 0.35**▵	−0.40 ± 0.37	0.932
	nD-eye	−1.03 ± 1.07	−0.67 ± 0.51	0.130	−0.69 ± 0.72	**−0.24 ± 0.20**▵	**<0.001**	**−0.49 ± 0.37**▵	−0.32 ± 0.35	0.140
SE (D)	D-eye	**−11.75 ± 3.03**	**−7.12 ± 2.42**	**<0.001**	−0.04 ± 0.29*▵	−0.06 ± 0.25*▵	0.934	−0.54 ± 0.39*▵	−0.29 ± 0.66*▵	0.443
	nD-eye	**−12.49 ± 3.47**	**−7.85 ± 3.30**	**<0.001**	−0.49 ± 0.57*▵	−1.35 ± 0.83*▵	**<0.001**	−1.15 ± 0.58*▵▴	−1.54 ± 0.74*▵	**0.014**
CDVA (Logmar)	D-eye	**0.04 ± 0.08**	**−0.02 ± 0.05**	**0.008**	**−0.05 ± 0.06**▵	−0.03 ± 0.05	0.252	**−0.06 ± 0.09**▵	−0.04 ± 0.10	0.539
	nD-eye	**0.05 ± 0.11**	**−0.01 ± 0.07**	**0.018**	**−0.03 ± 0.07**▵	−0.02 ± 0.07	0.740	**−0.01 ± 0.13**▵	0 ± 0.11	0.763
ADD (D)		**0.64 ± 0.29**	**1.25 ± 0.67**	**0.001**	NA	NA	NA	**1.33 ± 0.55**▵	**1.84 ± 0.78** ▵	**0.021**
Safety indices	D-eye	NA	NA	NA	**1.27 ± 0.22**	**1.03 ± 0.08**	**<0.001**	**1.29 ± 0.29**	**1.06 ± 0.19**	**0.001**
	nD-eye				**1.23 ± 0.26**	**1.04 ± 0.08**	**<0.001**	**1.19 ± 0.24**	**1.01 ± 0.21**	**0.015**
Efficacy indices	D-eye	NA	NA	NA	**1.25 ± 0.24**	1.01 ± 0.11*	**0.001**	**0.86 ± 0.29**▴	0.88 ± 0.27*▴	0.812
	nD-eye				**1.10 ± 0.33**	0.64 ± 0.25*	**<0.001**	**0.68 ± 0.28**▴	0.45 ± 0.25*▴	**0.006**

ICL, implantable collamer lens; FS-LASIK, femtosecond laser-assisted laser in situ keratomileusis; D-eye, dominant eye; nD-eye, nondominant eye; UDVA, uncorrected distance visual acuity; ADD, presbyopic add power; SE, spherical equivalent; CDVA, corrected distance visual acuity. ▵ versus Preoperative, *p* < 0.05. ▴ versus the 1-month follow-up, *p* < 0.05. * dominant eye versus nondominant eye, *p* < 0.05. Values with statistical significance are shown in bold.

**Table 3:** The biometric parameters before and after the implantable collamer lens V4c implantation or femtosecond laser-assisted laser *in situ* keratomileusis.

Parameters	Preoperative / 1-mon follow-up (Vault)			48-mon follow-up		
	ICL V4c Group	FS-LASIK Group	*P*	ICL V4c Group	FS-LASIK Group	*P*
ACD (mm)	**3.16 ± 0.35**	**2.98 ± 0.22**	**0.001**	**2.87 ± 0.34**▵	**2.85 ± 0.21**▵	0.728
ACV (μl)	180.89 ± 36.91	167.70 ± 26.46	0.177	**110.06 ± 24.82**▵	**156.04 ± 26.98**▵	**<0.001**
ACA (°)	36.55 ± 5.98	34.94 ± 5.13	0.083	**21.64 ± 4.39**▵	**33.51 ± 4.87**▵	**<0.001**
IOP (mmhg)	15.10 ± 2.31	16.05 ± 2.72	0.085	**16.13 ± 2.21**▵	**11.36 ± 2.03**▵	**<0.001**
AL (mm)	**28.69 ± 2.09**	**26.54 ± 1.33**	**<0.001**	**28.72 ± 2.04**▵	**26.66 ± 1.37**▵	**<0.001**
ECD (cell/mm^2^)	3107.31 ± 585.84	NA	NA	**2557.89 ± 290.98**▵	NA	NA
Vault (μm)	518.06 ± 196.13	NA	NA	**432.65 ± 208.03**▴	NA	NA

ACD, anterior chamber depth; ACV, anterior chamber volume; ACA, anterior chamber angle; IOP, intraocular pressure; ECD, endothelium cell density. ▵Versus preoperative, *p* < 0.05. ▴Versus the 1-month follow-up, *p* < 0.05. Values with statistical significance are shown in bold.

### 3.2. Monovision vision

**Figure 3:**
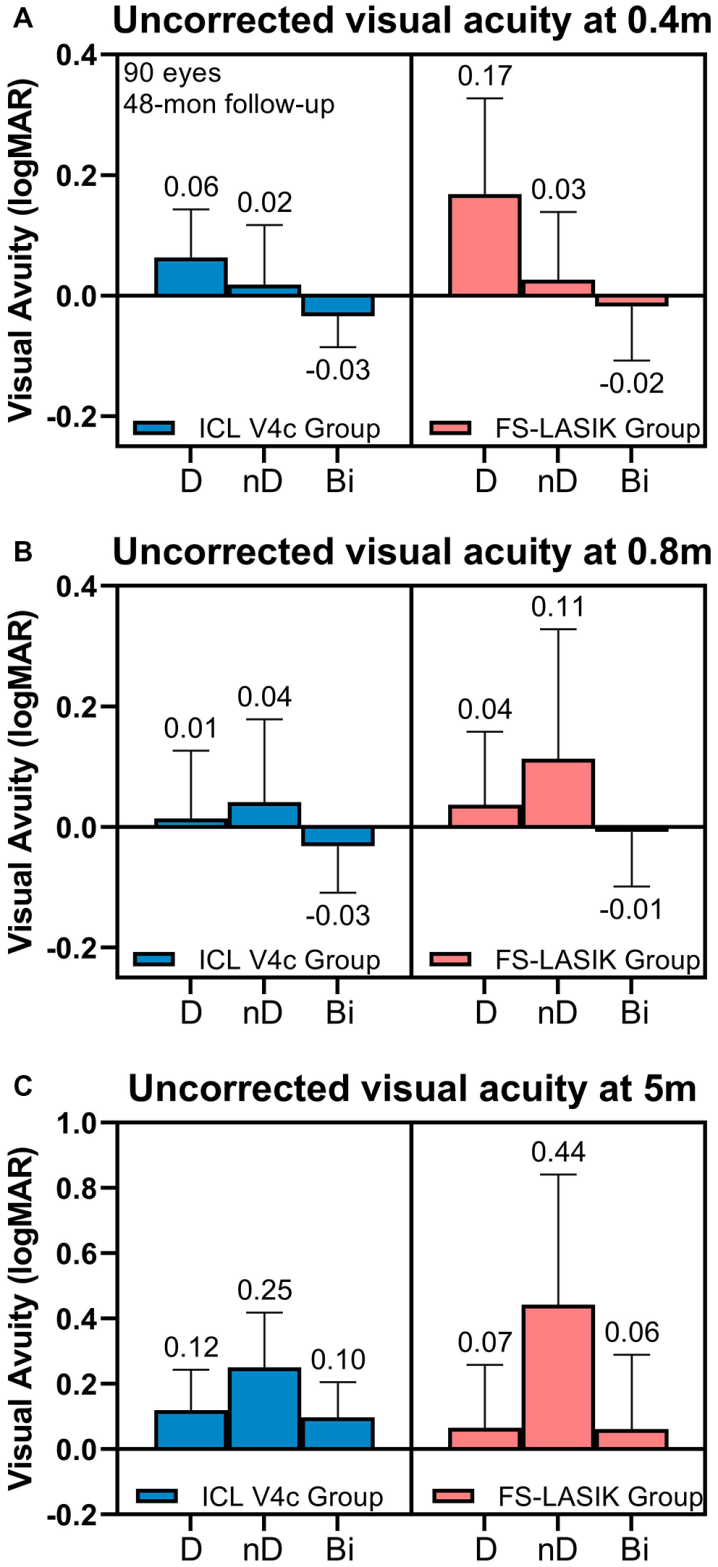
Uncorrected Visual Acuity at 0.4 m **(A)**, 0.8 m **(B)**, and 5.0 m **(C)** at 48 months after Implantable collamer lens V4c implantation or femtosecond laser-assisted laser *in situ* keratomileusis.

The binocular visual acuity (logMAR) of the ICL V4c group were −0.03 ± 0.05 (0.4 m), −0.03 ± 0.02 (0.8 m), and 0.10 ± 0.03 (5 m). The corresponding figures were −0.02 ± 0.09, −0.01 ± 0.02, and 0.06 ± 0.04 in the FS-LASIK group. The two groups had no significant difference (*p* > 0.05; Figure 3). The percentages of binocular VA > 20/25 (Snellen Line) at the three distances were 78 and 85.19% in the ICL V4c and FS-LASIK groups, respectively. The percentage of non-dominant eyes with VA > 20/25 (Snellen Line) at 0.4 m was 88.89 and 85.19% for the ICL V4c and FS-LASIK groups, respectively; the percentage with VA > 20/32 (Snellen Line) was 100 and 96.30% for the two groups, respectively. At 5 m distance, for the ICL V4c and FS-LASIK groups, those with VA > 20/25 (Snellen Line) were 76.47 and 81.48%, respectively, and VA > 20/32 (Snellen Line) were 2.35 and 88.89%, respectively.

### 3.3. Binocular balance

Table 4 shows the binocular balance of both groups at various distances. The proportion of patients with binocular imbalance (balance) at near, intermediate, and distances were 68.89% (31.11%), 71.11% (28.89%), and 82.22% (17.78%), respectively. The difference in binocular imbalance and its level between the two groups was not significant. Additionally, the binocular balance within each group at different distances showed no significant difference. At 0.4 m, the proportions of patients with binocular balance levels 1–5 (contrast sensitivity) were 2.22, 6.67, 11.11, 4.44, and 6.67%, respectively; at 0.8 m, they were 4.44, 6.67, 11.11, 0.00, and 6.67%, respectively; and at 5.0 m, they were 2.22, 4.44, 4.44, 6.67, and 0.00%, respectively.

**Table 4:** Binocular imbalance in patients 48 months after implantable collamer lens V4c implantation or femtosecond laser-assisted laser *in situ* keratomileusis.

Distance	Characteristic	ICL V4c Group	FS-LASIK Group	*p*
0.4 m	Balanced/Imbalanced	8/10	6/21	0.595
	Level of balance	1.50 ± 1.95	0.67 ± 1.36	0.454
	Range of balance (D-eye)	3.06 ± 1.63	2.93 ± 1.27	0.721
	Range of balance (nD-eye)	2.44 ± 1.76	2.93 ± 1.17	0.078
0.8 m	Balanced/Imbalanced	7/11	6/21	0.760
	Level of balance	1.11 ± 1.75	0.67 ± 1.36	0.665
	Range of balance (D-eye)	2.67 ± 1.61*	2.81 ± 1.27	0.085
	Range of balance (nD-eye)	1.89 ± 2.00*	**2.96 ± 1.19**	**0.025**
5.0 m	Balanced/Imbalanced	5/13	3/24	0.296
	Level of balance	0.89 ± 1.53	0.26 ± 0.86	0.233
	Range of balance (D-eye)	1.50 ± 1.69	1.37 ± 1.84	0.891
	Range of balance (nD-eye)	1.00 ± 1.33	0.74 ± 1.53	0.687

*Dominant eye versus nondominant eye, *p* < 0.05. Values with statistical significance are shown in bold.

### 3.4. Contributing factors to binocular balance

#### 3.4.1. Near distance (0.4 m)

**Figure 4:**
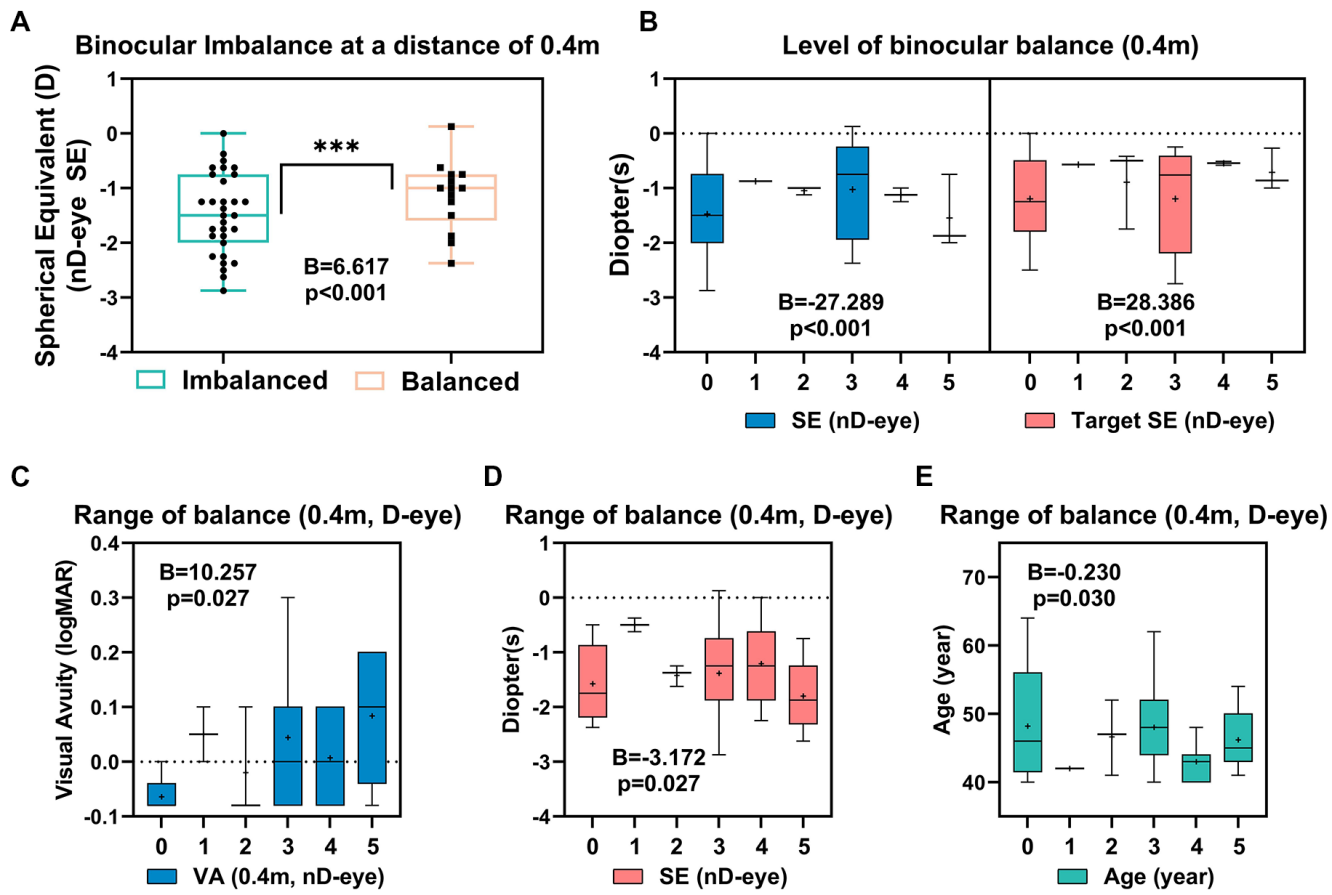
Correlated parameters of binocular imbalance **(A)**, level of binocular balance **(B)** and the range of balance **(C–E)** at 0.4 m.

The SE of non-dominant eye (−1.14 ± 0.17D) for patients with binocular balance at 0.4 m significantly differs from that with binocular imbalance (−1.47 ± 0.13D) (*B* = 6.617, *p* < 0.001; Figure 4A). The degree of anisometropia between the non-dominant and dominant eyes did not differ between balanced and imbalanced patients (*B* = −1.446, *p* = 0.145). The binocular balance level was related to the monovision design and SE of the non-dominant eye at the last follow-up (*B* = −27.289, *p* < 0.001; *B* = 28.386, *p* < 0.001) (Figure 4B). The range of balance of the dominant eye may be related to the SE of the non-dominant eye (*B* = 10.257, *p* = 0.027) (Figure 4C), visual acuity at 0.4 m (*B* = −3.172, *p* = 0.027) (Figure 4D), and age (*B* = −0.230, *p* = 0.030) (Figure 4E).

#### 3.4.2. Middle distance (0.8 m)

**Figure 5:**
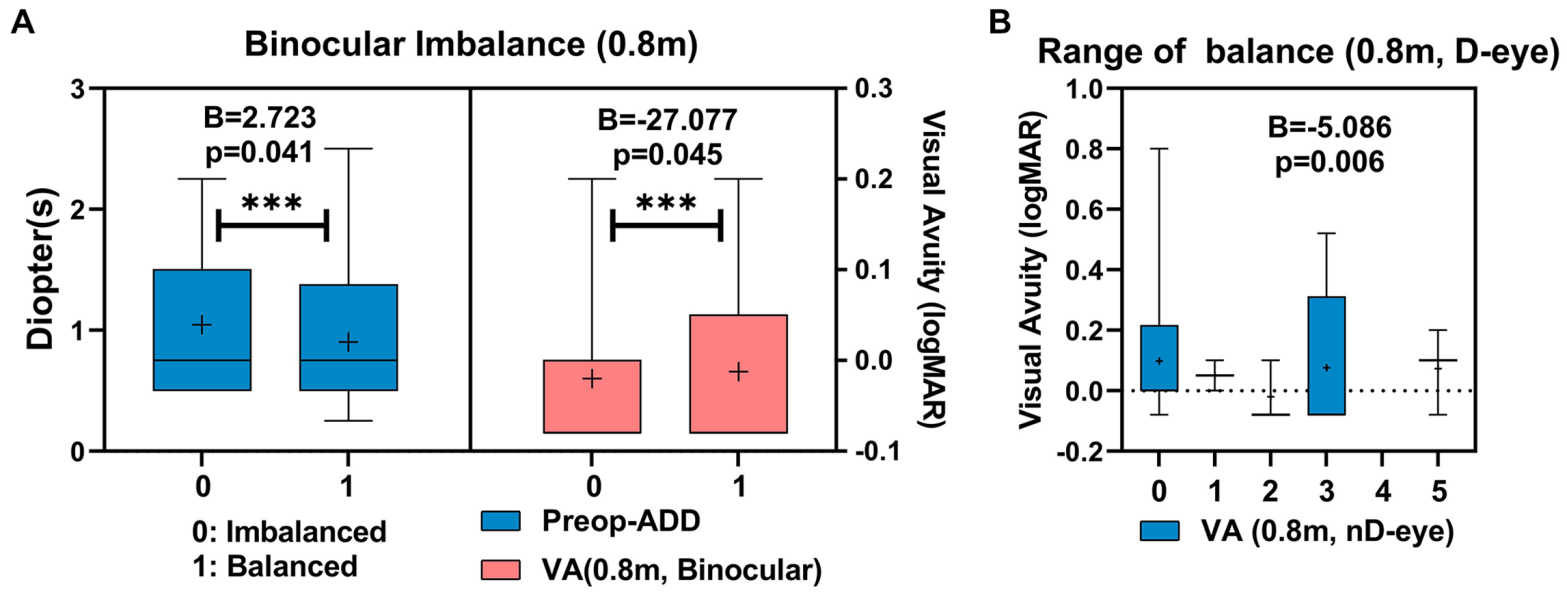
Correlated parameters of binocular imbalance **(A)**, and the range of balance **(B)** at 0.8 m.

There was a significant difference in preoperative ADD between the binocular balanced (0.90 ± 0.17D) and the imbalanced (1.05 ± 0.11D) patients at 0.8 m (*B* = 2.72, *p* = 0.041). Additionally, there was a significant difference in binocular visual acuity at 0.8 m between the binocular balanced patients (−0.01 ± 0.03) and the imbalanced (−0.02 ± 0.01D) at 0.8 m (*B* = −27.077, *p* = 0.045; Figure 5A) As shown in Figure 5B, The range of balance in the dominant eye might be correlated to the binocular visual acuity at 0.8 m of the non-dominant eye (*B* = −5.086, *p* = 0.006).

#### 3.4.3. Far distance (5.0 m)

**Figure 6:**
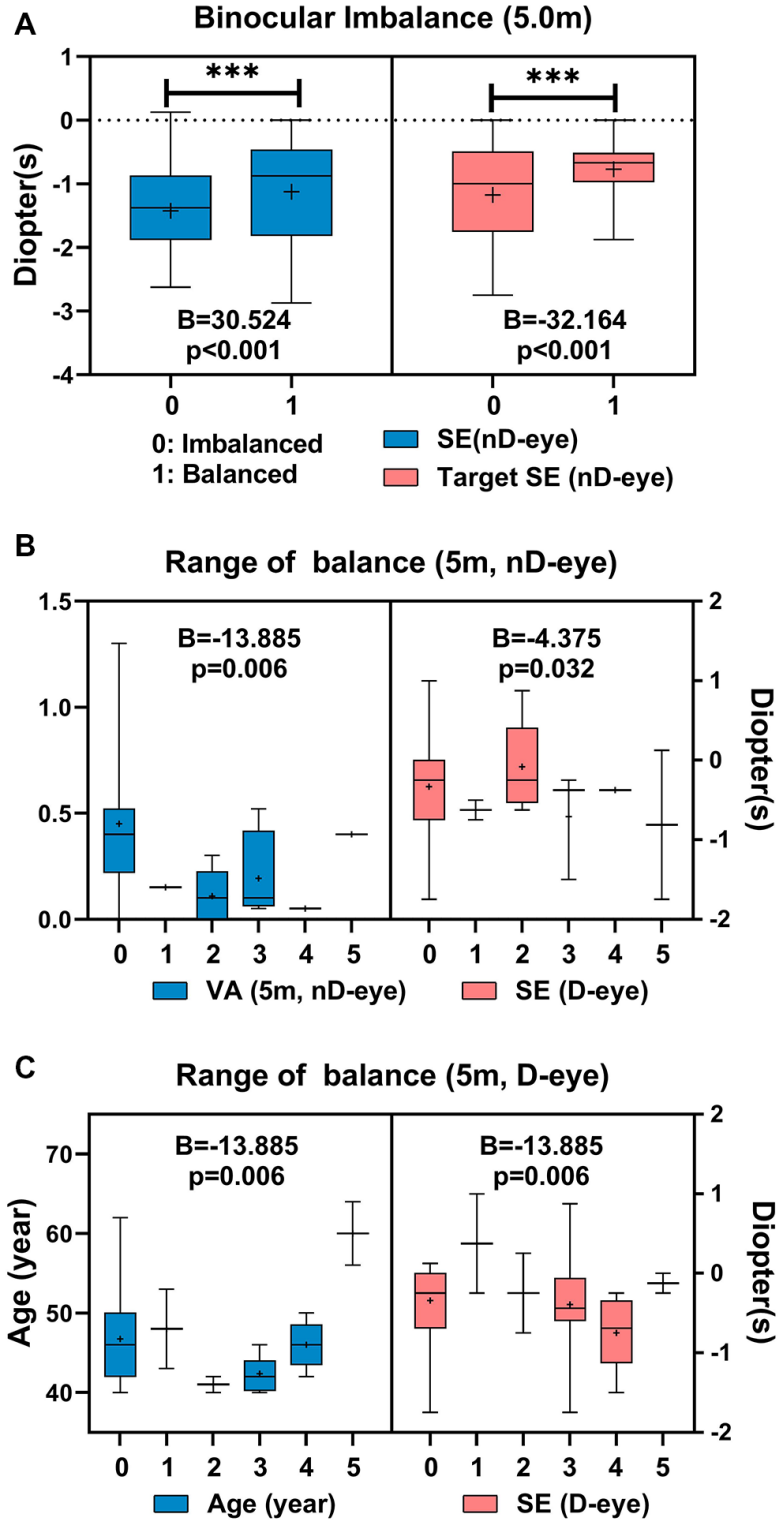
Correlated parameters of binocular imbalance **(A)**, and the range of balance **(B,C)** at 5 m.

There was a significant difference in SE of the non-dominant eye between the binocular balanced patients (−1.13 ± 0.33D) and the imbalanced (−1.42 ± 0.11D) at 5 m (*B* = 30.524, *p* < 0.001). Additionally, there was a significant difference in target SE of non-dominant eye between the binocular balanced patients (−0.78 ± 0.19D) and the imbalanced (−1.17 ± 0.13D) at 5.0 m (*B* = −32.164, *p* < 0.001; Figure 6A). As shown in Figure 6B, The range of balance in the non-dominant eye might be correlated to the visual acuity of the non-dominant eye at 5.0 m (*B* = −13.89, *p* = 0.006) and the real refraction of the dominant eye (*B* = −4.375, *p* = 0.032). The range of balance of the dominant eye may be correlated with age (*B* = 0.386, *p* = 0.002) and the visual acuity of the dominant eye at 5.0 m (*B* = −2.291, *p* = 0.036) (Figure 6C).

## 4. Discussion

This study elucidated the safety index of the two surgical procedures after 4 years postoperatively, which was 1.24 ± 0.27 and 1.04 ± 0.20, for the ICL V4c and FS-LASIK groups, respectively, while the efficacy index was 0.77 ± 0.29 and 0.66 ± 0.34, respectively. The efficacy index was lower owing to intentional undercorrection in the non-dominant eye than in conventional studies. However, considering good binocular visual acuity at near-to-far distances, monovision surgery using ICL V4c or FS-LASIK has good long-term safety and efficacy in myopic patients with presbyopia. With the increasing number of myopic patients and the aging of the population, the safety and efficacy of refractive surgery in people aged ≥40 years have received widespread attention (Levinger et al., 2013; Kamiya et al., 2017; Primavera et al., 2020; Tañá-Rivero et al., 2020). The safety of ICL V4c implantation and FS-LASIK surgery in this population has been validated through comprehensive evaluations of multiple aspects, such as lens density (Ye et al., 2021) and monovision surgery for treating myopia combined with presbyopia (Kamiya et al., 2017; Takahashi et al., 2018; Ye et al., 2022). However, there has been little discussion on long-term effects. These results were similar to those after three years following monovision surgery using ICL V4c (1.22 ± 0.18 and 0.85 ± 0.29; Ye et al., 2022). Moreover, regarding biological parameters, ICL V4c implantation also exhibited long-term safety, with a similar decline in endothelial cell density to that reported in previous studies (Ye et al., 2021). The vault remained within a safe range (Gonvers et al., 2003).

Anisometropia between the dominant and non-dominant eyes caused by monovision may affect the visual function, which may affect their visual experience. A study has shown that anisometropia may lead to binocular imbalance and accommodative difficulties (Yang et al., 2017). This imbalance can cause the patients’ vision to deteriorate at near or far distances, affecting visual comfort and quality. This study evaluated the balance of binocular vision and found that imbalances were common in this population at near, intermediate, and distant distances. Previous studies have shown that the binocular visual acuity of this population is often best at intermediate distances and that the dominant and non-dominant eyes have the largest overlap at this distance, making binocular vision at this distance have the greatest impact on binocular balance (Takahashi et al., 2018; Ye et al., 2022).

This study showed that at different distances, the balanced range of the dominant eye was related to the visual acuity or SE of the non-dominant eye and also showed a correlation with age at far and near distances. The correlation of visual acuity was that the closer the binocular visual acuity, the higher the balance range. The correlation between the dominant eye’s balance range and the non-dominant eye’s SE showed the opposite result. Thus, the higher the degree of under-correction of the non-dominant eye at near distances, the lower the degree of under-correction at far distances, and the higher the balance level of the dominant eye. This contrasts the extreme relationship between this parameter and eye balance. Previous studies have suggested that an increase in monocular blur may increase the stereo threshold at high spatial frequencies (Li et al., 2016) and that the increase in threshold may be related to suppression under binocular imbalance (Zheleznyak et al., 2015). An “adaptation phenomenon” in the binocular balance under monovision is speculated, which may increase binocular imbalance and the range of balance of the dominant eyes in the balanced eyes. This adaptation of the non-dominant eye after suppression indicates the potential feasibility of visual function training in this population.

People commonly experience binocular imbalance, with 65.9 and 62.89% experiencing low-and high-temporal-frequency stimuli, respectively (Xu et al., 2019). The proportion of binocular imbalance in this study at a 0.4 m distance was similar at 68.89%, while there was a certain degree of increase in the imbalance at intermediate and far distances (71.11 and 82.22%, respectively). Previous studies have demonstrated significant binocular balance and acuity differences between older individuals and those with myopia (Arani et al., 2019). It has been suggested that the binocular competition rate is lower in older individuals than in young people (Vera-Diaz et al., 2018), while binocular imbalance is more pronounced in myopic patients than in the emmetropic population. Therefore, this study’s proportion of subjects with binocular imbalance may have been higher than that of age-matched individuals because of monovision. In simulated experiments in patients with anisometropia, visual acuity distribution at different distances from the dominant eye was an important factor affecting the contrast threshold performance (Zheleznyak et al., 2015). The increase in the imbalance proportion at intermediate and far distances may be related to changes in the contrast threshold or the gradual emergence of binocular imbalance with increasing testing time (Xu et al., 2019). Based on studies on neural rhythms, binocular imbalance may manifest as periodic changes in monocular dominance and dynamic fusion perception. Further research is necessary to determine whether these periodic changes are characteristic of monovision surgery. Treatment regimens that reduce suppression by promoting exposure to balanced binocular stimulation have improved visual acuity and stereo sensitivity (Kwon et al., 2014). Training in binocular balance stimulation after monocular design surgery may help further improve its efficacy.

Research conducted among myopic adolescents aged 6–18 years showed that an increase in binocular imbalance does not necessarily imply poorer SE or VA. Instead, an increase in anisometropia significantly correlated with an increase in binocular imbalance, and the dominant eye tended to have a more negative SE than the non-dominant eye (Tao et al., 2022). This study found that the balance of binocular vision at different distances was related to the refraction and visual acuity of both the dominant and non-dominant eyes. Unlike previous studies, this study showed no direct correlation between binocular balance and anisometropia. At a near distance of 0.4 m, the binocular balance was related to the SE of the non-dominant eye, while the balance threshold of the dominant eye depended on the non-dominant eye’s worse visual acuity at a near distance. Therefore, the difference in results between the two studies at a near distance suggests that the balance vision of patients with myopia and presbyopia after monovision surgery is significantly related to the SE of the non-dominant eye under a monovision design compared with adolescents. A more negative SE in the non-dominant eye implies a more severe binocular imbalance. The more negative SE of the dominant eye (from the perspective of binocular balance) in adolescents may be related to the fact that the measurements were conducted at a near distance. Consequently, it suggests that binocular balance is not only a common and dynamic process (Xu et al., 2019) but is also closely related to the testing distance (Zheleznyak et al., 2015) and its corresponding accommodation.

This study had certain limitations. First, the preoperative binocular balance was not compared. However, with the advancement and optimization of detection methods for binocular balance, future studies can better evaluate the changes before and after monovision surgery. The short-term plasticity of visual perception training can also be added to increase data variability before and after training, which can provide valuable clinical support. Second, tests for stereo vision function were not applied in this study, and future research is needed as binocular imbalance and stereo vision are two different aspects of binocular interactions. Previous studies have shown that subjects with good binocular balance tend to have better stereo vision. Therefore, studying different aspects of binocular relationships, such as stereo vision, in this population will contribute to further evaluating the clinical effects of monovision surgery. Third, the binocular imbalance was not measured during different working hours, and transient binocular imbalance is likely a temporary physiological phenomenon (Xu et al., 2019). As time progresses, older adults may experience longer balance times and lower alternation probabilities than younger people (Arani et al., 2019; Wang et al., 2021). The binocular imbalance under different work lengths after monovision surgery may vary. Further research in this area will help assess functional defects, such as reading disorders, under prolonged binocular imbalance conditions. Fourth, this study had a relatively small sample size, and larger sample studies are required to evaluate binocular balance function. The proportions of two subpopulation were different, further researches are warranted including comparable subgroup cases.

In conclusion, the ICL V4c implantation and FS-LASIK monovision treatment demonstrated good long-term safety and binocular visual acuity at various distances. The imbalanced patients’ vision is primarily related to the age-related presbyopia and anisometropia progression caused by the monovision design. High levels of anisometropia may required careful consideration from the aspect of biocular balance.

## Data availability statement

The raw data supporting the conclusions of this article will be made available by the authors, without undue reservation.

## Ethics statement

This study followed the tenets of the Declaration of Helsinki and was approved by the Ethics Committee of Fudan University Eye and ENT Hospital Review Board (Shanghai, China; ID: 2013015, date: 2013/1/5). Informed consent was obtained from all participants. The patients/participants provided their written informed consent to participate in this study.

## Author contributions

YY, ZZ, JZ, and XZ: study concept and design. YY, ZZ, LN, WS, XW, and JZ: data collection. YY: data analysis and interpretation. YY and JZ: drafting of the manuscript. LY, JZ, and XZ: critical revision of the manuscript. XZ: supervision. All authors read and approved the final manuscript.

## Funding

This study was supported by National Natural Science Foundation of China (Grant no. 82271119), Shanghai Rising-Star Program (23QA1401000), Healthy Young Talents Project of Shanghai Municipal Health Commission (2022YQ015), Project of Shanghai Science and Technology (Grant nos. 20410710100 and 21Y11909800), and Project of Shanghai Xuhui District Science and Technology (2020-015).

## Conflict of interest

The authors declare that the research was conducted in the absence of any commercial or financial relationships that could be construed as a potential conflict of interest.

## Publisher’s note

All claims expressed in this article are solely those of the authors and do not necessarily represent those of their affiliated organizations, or those of the publisher, the editors and the reviewers. Any product that may be evaluated in this article, or claim that may be made by its manufacturer, is not guaranteed or endorsed by the publisher.

## Footnotes

^1^ http://en.informer.com/icl-power-calculation-software/
