# Supplementary figures and images for: Binocular imbalance in patients after implantable collamer lens V4c implantation or femtosecond laser-assisted in situ keratomileusis for myopia with presbyopia
Source: Front Neurosci. 2023 Jun 1;17:1204792. doi: 10.3389/fnins.2023.1204792 (PMC10267309; doi:10.3389/fnins.2023.1204792)

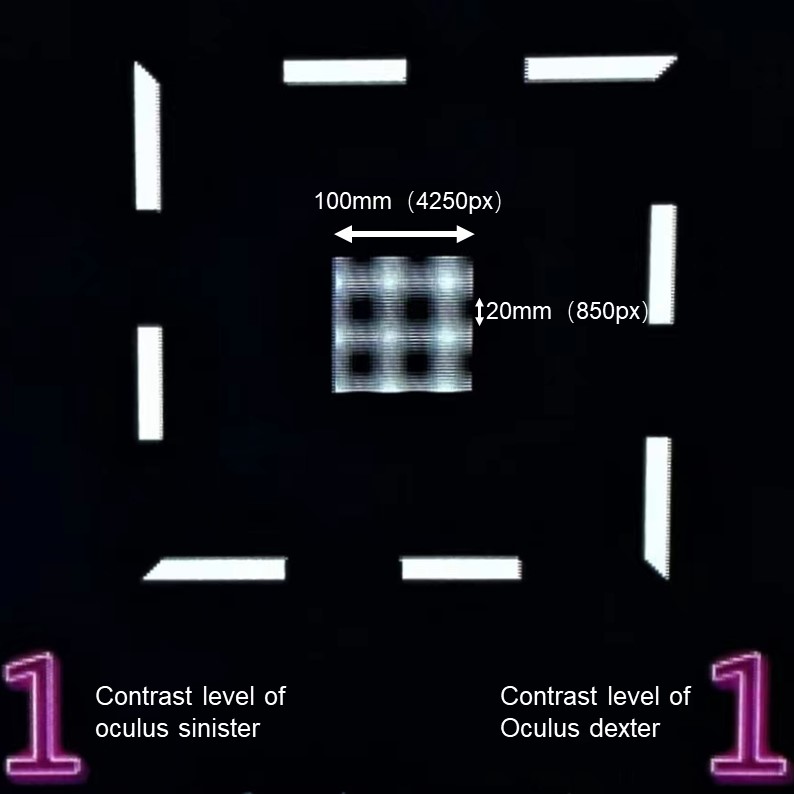

Supplement: Supplementary file 1 [file Image_1.JPEG]
